# Clinicopathological characteristics and health care for Tibetan women with breast cancer: a cross-sectional survey

**DOI:** 10.1186/s12885-019-5580-x

**Published:** 2019-04-25

**Authors:** Yulan Zhao, Hanhuan Luo, Xintian Zhang, Tashi Bianba, Lin Li, Qian Wang, Lei Guo, Dian Wang, Yongge Ze, Shan Zheng

**Affiliations:** 1 Department of Oncology, Tibet autonomous region people’s hospital, autonomous region, Lhasa, 850000 Tibet China; 2 Department of Pathology, Tibet autonomous region people’s hospital, autonomous region, Lhasa, 850000 Tibet China; 3 Department of Second Clinical Medicine, Sun Yat-sen University Zhongshan School of Medicine, Yuexiu District, Guangzhou, 510080 Guangdong China; 4 Department of Pathology, National Cancer Center/National Clinical Research Center for Cancer/Cancer Hospital, Chinese Academy of Medical Sciences and PeKing Union Medical College, Beijing, 100021 China; 5 Department of Imaging, Tibet autonomous region people’s hospital, autonomous region, 850000 Tibet, China

**Keywords:** Breast cancer, Clinicopathological characteristics, Tibetan female, Plateau, Healthcare system

## Abstract

**Background:**

The healthcare system (HCS) improved in Tibet Autonomous Region (TAR), China. The present study aimed to investigate whether these improvements might alter the clinicopathological characteristics of a Tibetan female with breast cancer (BC) in TAR.

**Methods:**

This was a single-center cross-sectional study conducted at TAR People’s Hospital. All Tibetan adult women were treated for BC in this hospital between January 1, 1973 and December 31, 2015. The inclusion criteria were as follows: (1) Tibetan adult woman living in Tibet; (2) Histopathology or cytopathology or both confirming primary BC; (3) All the treatments were finished in this hospital. χ^2^ test and logistic regression were applied, using age group and census register as the two covariates.

**Results:**

A total of 273 patients with BC were included in the final analysis. Of these, 14 patients were in the free HCS, 183 patients had medical insurance combined with a new rural cooperative HCS, and 76 were in a rural and urban integration HCS. Currently, a rural and urban integration HCS is an improved system. Consequently, an increase in the proportion patients in the T1–3 stage was observed (0.198; 0.046 to 0.852) between the rural and urban integration HCS and free HCS. The proportion of patients in early (I + II) stage cancer (0.110; 0.019–0.633) also increased between these two HCSs.

**Conclusion:**

This was the first report about Tibetan women with BC in Tibet. Some clinicopathological characteristics at the presentation of Tibetan women with BC may improve during different HCSs. The cancer awareness, early detection, and the overall management in patients with advanced stage BC might improve the prognosis of BC in the rural and urban integration HCS.

## Background

The data of International Agency for Research on Cancer illustrated that breast cancer (BC) is major cancer in female worldwide since 1975 [1–5]. In China, the rank of BC on the incidence list was the same as that in the world since 2004, while the rate of mortality ranked in the list of top ten causes [6–8]. Strikingly, BC is one of the diseases related to the level of the health -care system (HCS) [9–11]. The aggressive nature of BC in a restricted HCS might be attributed to low cancer awareness, high body weight index, less physical activity, less access to screening, poor population-based cancer registries, and less research on these tumors. Furthermore, after diagnosis, BC is usually in later stage with comorbidity, less treatment choices and *etc* [12–15].

Tibet Autonomous Region (TAR), known as “the roof of the world” with an average altitude above 4000 m, lies in southwest frontier of China under a relatively restricted level of HCS in China [16]. Tibetan nationality is one of the Chinese minorities in this anoxic plateau for centuries. Since the anoxic plateau was not suitable for most of the nationalities except Tibetan, the population features in this nationality showed relative stability, such as similar lifestyle, same religion, breastfeeding, and ≥ 2 children during these centuries [16]. Nowadays, the level of HCS has been improved greatly. For example, the screening of female BC in TAR counties has been conducted since 2009 according to the national guidelines of China [17, 18]. We hypothesized that the clinicopathological characteristics of the Tibetan female with BC might be aggressive as that in the other regions with fewer HCSs [12]. The improvement in HCS may improve some characteristics. In this study, we described, for the first time, the clinicopathological characteristics at the presentation of Tibetan female with BC, and then, identified the influence of different HCSs on these characteristics.

This is the first report of the clinicopathological characteristics at the presentation of Tibetan women with BC in TAR, initiated by the two main hospitals in Tibet and Beijing, China. Herein, we focused on the shift of the clinicopathological characteristics and discussed the underlying reasons for this shift. These data might provide valuable insight and recommendations for the related agencies in China, as well as, for female BC in restricted HCS in other countries.

## Methods

### Study design and quality control

A cross-sectional study was conducted from TAR People’s Hospital and focused on Tibetan women with primary BC that formed a cohort in the study of Tibetan women with primary BC. We collected all Tibetan women with BC in this hospital, from inception to date. This study had approved by the review of the Ethics Committee of TAR People’s Hospital (ID Num: ME-TBHP-15-1). Patient consent was not required according to the routine. The data were stripped of any patient identifiers as per the approved procedures and maintained in secure database. In this study, we discussed the association of different HCSs with the clinicopathological characteristics at presentation.

All patients enrolled in this study must fulfill three key inclusion criteria: (*i*) Tibetan women lived in TAR; (*ii*) histopathology and/or cytopathology confirmed primary BC; (*iii*) the comprehensive treatment model (surgery, medical oncology and endocrine therapy) was finished in this hospital.

Data from all cases were collected with quality control: utilizing proper case report forms to extract the information, local clerk training, double-enter of data, validation by EpiData and consistency check.

*Data collection and stratification.* Primary medical reports were extracted, including demographic information, risk factors, symptoms and signs, imaging and laboratory tests, medical history, therapeutic models and pathologic characteristics. The histopathology data were reviewed by Zheng, Luo and Wang, according to 2012 WHO breast tumor histological classification criterion [19]. The tumor Stage of each patient was reviewed by Ze, Zhao and Bianba, according to 2010 American Joint Committee on Cancer (AJCC) TNM stage criterion [20].

**Fig. 1:**
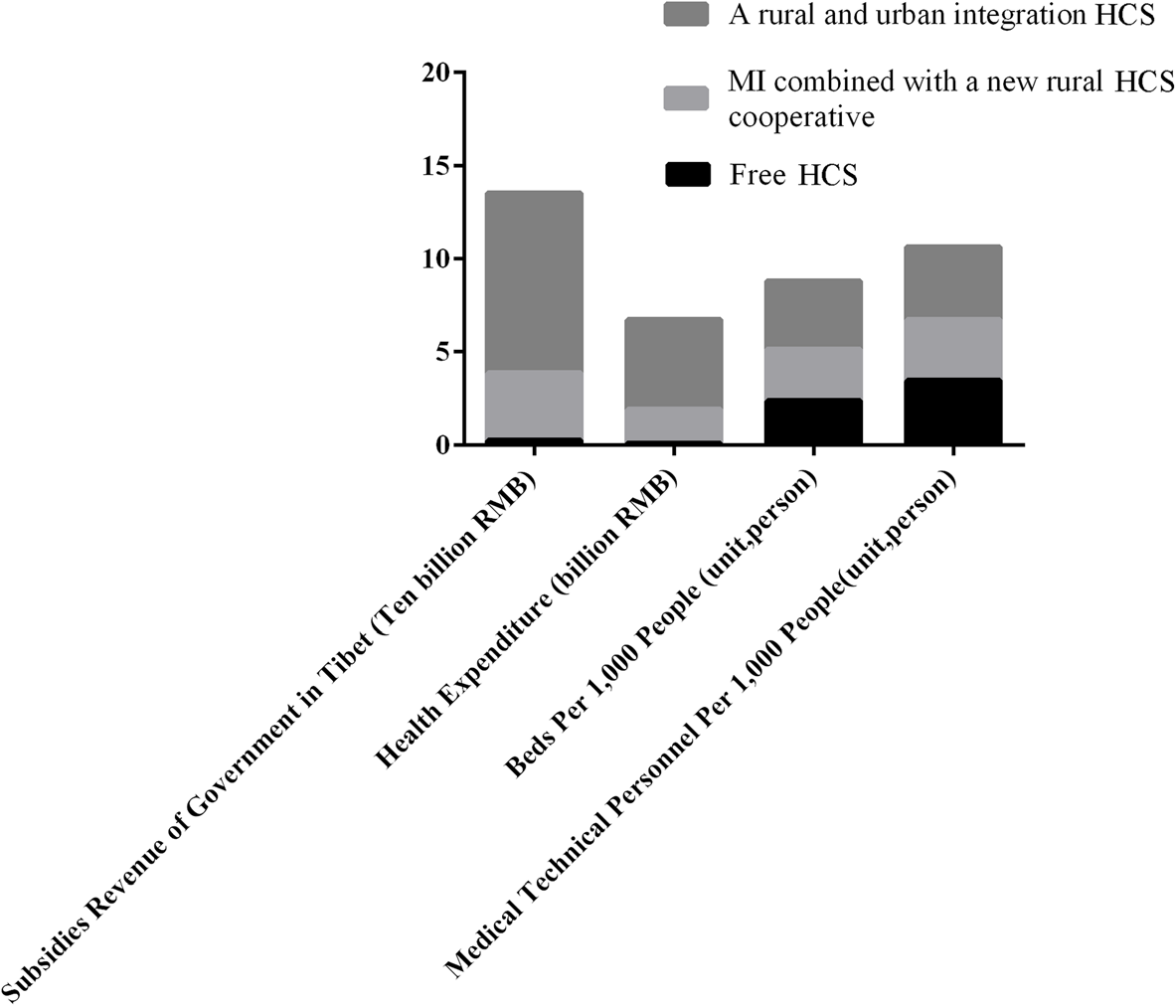
Comparison of different healthcare system in TAR. Black bars represent data in free HCS. Light grey bars represent data in MI combined with a new rural cooperative HCS. Heavy grey bars represent data in a rural and urban integration HCS. The subsidies by government and health expenditures had improved greatly, while the condition in beds per 1000 people and medical technical personnel per 1000 people were relative stable in the three different HCSs

TAR had three different HCSs according to the characteristics of four major dependent factors of HCS (Fig. 1): subsidies revenue of government in Tibet, health expenditure, beds and medical technical personnel per 1000 people [16, 17, 21–23]. Free HCS, from the hospital’s foundation day to 2001, showed a low level of these four major dependent factors. Medical insurance (MI) combined with a new rural cooperative HCS, from 2002 to 2012, showed that the level of these four major dependent factors was improved. A recent rural and urban integration HCS, from 2013 to 2015, showed that the level of these four factors improved greatly.

*Statistical analysis.* The major characteristics of invasive BC, including tumor size, pathologic type and grade, lymph node metastasis (LNM), T stage and AJCC stage were investigated. The cases with missing cases were excluded. Tumor size was divided into two groups of ≤2 cm and > 2 cm. The pathologic type was divided into two groups of Non special type (NOS) and special type. The Nottingham combined histology grade was divided into three groups of I, II and III. LNM was divided into two groups: “N1–3” vs. “N0”. The T stage was divided into two groups: “T1–3” vs. “T4”. The AJCC stages were divided into early (I + II) stage, local advanced (III) stage and advanced (metastatic or recurrence BC/IV) stage.

Categorical variables were analyzed by Spearman Chi-square test. The ordinal variables with more than two levels were analyzed by Cochran-Armitage Chi-square test. Subsequently, multivariate logistic regression was performed, using age group and census register as the two covariates. The age was divided into two groups of ≤50 and > 50 years, according to the mean age of these patients. The census register of the patient was divided into urban resident or farmers and herdsmen groups. Here, HCS and census register were used as proxy to the SES in our study. All missing information was not included in the analysis.

The statistical analyses were performed using the software SPSS 16.0 (SPSS Inc. Chicago, IL, USA). Statistical significance was assessed by two-tailed tests with *P* < *0.05* without adjustment for multiplicity, since all analyses were to be exploratory.

## Results

**Fig. 2:**
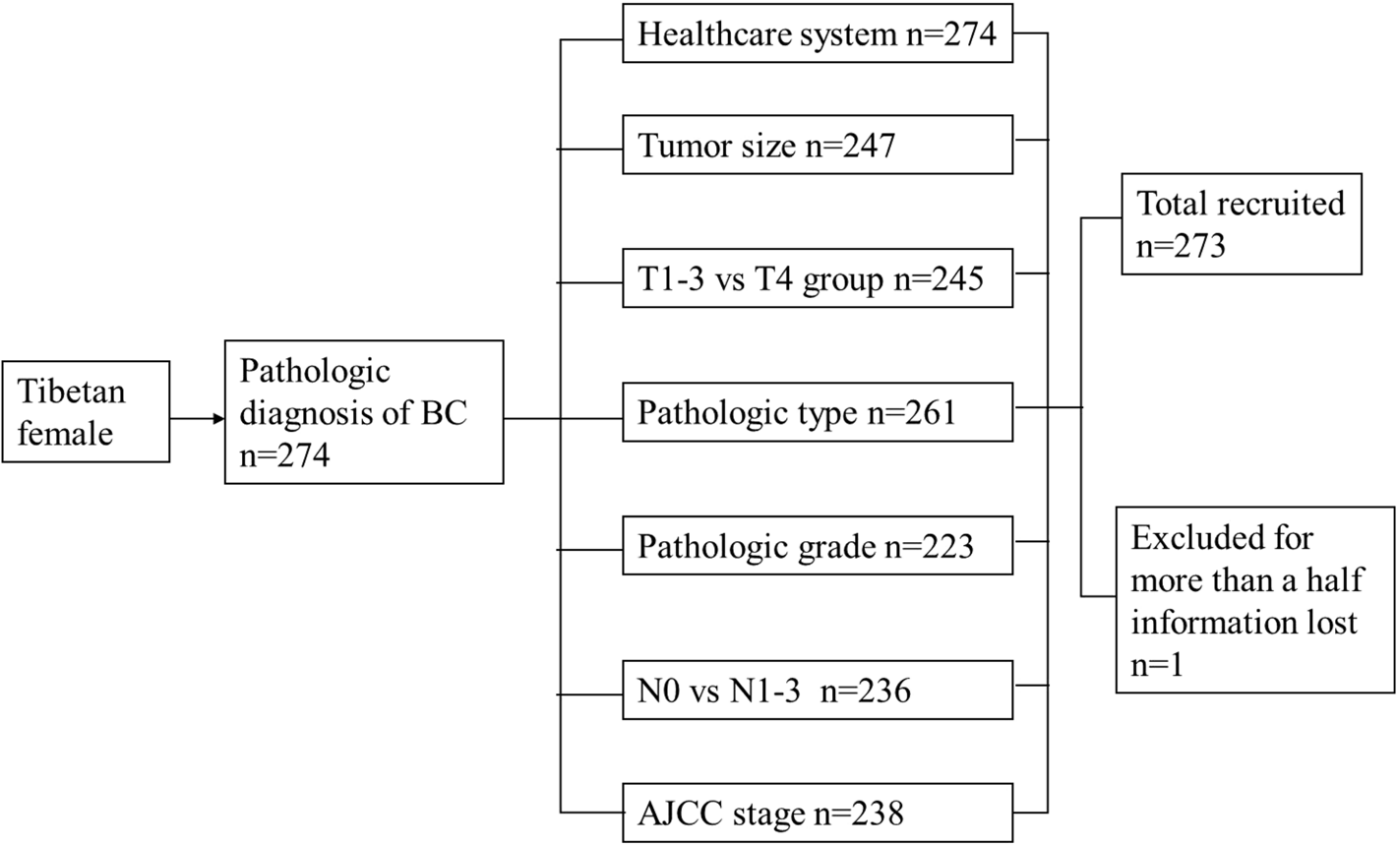
Flow diagram of Tibetan female with BC in TAR People Hospital. All the Tibetan female with BC first selected by pathologic diagnosis, then selected by HCS and clinicopathologic characters. Cases in more than a half information lost were excluded

We reviewed the index of all patients in TAR people’s hospital records from 1952 to 2015. The first case of Tibetan female patient with BC was admitted in 1973; she originated from Lhasa. Until Dec. 31, 2015, 274 cases of Tibetan female with BC had been received and treated at the hospital. Presently, of these, about 30 patients still visit this hospital per year. However, one case was excluded for final analysis due to loss of information (Fig. 2).

*Baseline data of 273 Tibetan female with BC.* The baseline data of the 273 Tibetan female with primary BC were listed in Table 1. The mean age of these patients was 50.4 years, and a majority was postmenopausal (44.7%). The cohort consisted of 14 Tibetan females with BC in free HCS, 183 in the MI combined with a new rural cooperative HCS, and 76 in a rural and urban integration HCS. Moreover, 95.6% (261/273) patients underwent the histopathologic test for a definite diagnosis, while 17.6% (48/273) patients were diagnosed by both histo- and cytopathology.

**Table 1:** Baseline information of 273 Tibetan female with primary breast carcinoma

Baseline information	Total
Age	
Mean ± SD	50.4 ± 10.6 (29,79)
Median	49.0
Age group	
≤ 50	155 (56.8)
> 50	118 (43.2)
Menstrual status	
Premenopause (*n*,%)	93 (34.1)
Postmenopausal (*n*,%)	122 (44.7)
Unknown (*n*, %)	58 (21.2)
Total (*n*, %)	273 (100.0)
Altitude	
< 4000 m (*n*, %)	101 (37.0)
≥ 4000 m (*n*, %)	61 (22.3)
Unknown (*n*, %)	111 (40.7)
Total (*n*, %)	273 (100.0)
Social economic status of area	
High (Lhasa) (*n*, %)	135 (49.5)
Low (other area except Lhasa) (*n*, %)	138 (50.5)
Total (*n*, %)	273 (100.0)
Census register	
Urban residents (*n*, %)	117 (42.8)
Farmers and herdsmen (*n*, %)	155 (56.8)
Unknown (*n*, %)	1 (0.4)
Total (*n*, %)	273 (100.0)
HCS	
Free HCS (*n*, %)	14 (5.1)
MI combined with a new rural cooperative HCS (*n*, %)	183 (67.0)
A rural and urban integration HCS (*n*, %)	76 (27.9)
Diagnostic model	
Cytopathology	60 (22.0)
Histopathology	261 (95.6)
Cytopathology+Histopathology	48 (17.6)
Total (*n*, %)	273 (100.0)

HCS = Health care system; MI = Medical insurance

*Clinicopathological characters of 273 Tibetan female with BC.* The proportion of patients whose tumor is >2 cm in diameter accounted for 83.5% (228/273). Of this, 27.8% (76/273) cases belonged to the T4 stage; only skin invasion was the most common type in T4 stage (23.4%, 64/273). Three patients were diagnosed with ductal carcinoma in situ (DCIS), while 220 patients were diagnosed with invasive BC, NOS (80.6%, 220/273). Most patients showed IIor III grade (72.2%, 197/273). The N1–3 group constituted 64.1% (175/273) of the cohort, while N2 was most common in this group. The main AJCC stage at diagnosis was stage III (50.5%, 138/273) (Table 2).

**Table 2:** Clinical and pathological characters of 273 Tibetan female with BC

Clinical characters	
Tumor size	
≤ 2 cm (*n*, %)	19(7.0)
>2 cm (*n*, %)	228(83.5)
Unknown (*n*, %)	26(9.5)
Total (*n*, %)	273(100.0)
T4 stage	
Skin invasion only (*n*, %)	64(23.4)
Chest invasion only (*n*, %)	6(2.2)
Skin and Chest invasion (*n*, %)	6(2.2)
- (*n*, %)	169(61.9)
Unknown (*n*, %)	28(10.3)
Total (*n*, %)	273(100.0)
Pathologic characters	
Pathologic type	
Ductal carcinoma in situ (*n*, %)	3(1.1)
Invasive carcinoma, Nos (*n*, %)	220(80.6)
Invasive carcinoma, special type (*n*, %)	25(9.1)
Invasive carcinoma, untyped	13(4.8)
BC, untyped	12(4.4)
Total (*n*, %)	273(100.0)
Pathologic grade	
Ductal carcinoma in situ	
High grade (*n*, %)	3(1.1)
Middle/Low grade (*n*, %)	0(0)
Invasive carcinoma, NOS	
I (*n*, %)	23(8.4)
II (*n*, %)	102(37.4)
III (*n*, %)	95(34.8)
Ungraded of invasive carcinoma, special type or untyped (*n*, %)	38(13.9)
Unknown (*n*, %)	12(4.4)
Total (*n*, %)	273(100.0)
LNM	
N1 (*n*, %)	57(20.9)
N2 (*n*, %)	80(29.3)
N3 (*n*, %)	38(13.9)
N0 (*n*, %)	61(22.3)
Unknown (*n*, %)	37(13.6)
Total (*n*, %)	273(100.0)
AJCC stage	
0 (*n*,%)	3 (1.1)
I(*n*,%)	3(1.1)
II (*n*, %)	78(28.6)
III (*n*, %)	138(50.5)
IV (*n*, %)	16(5.9)
Unknown (*n*, %)	35(12.8)
Total (*n*, %)	273(100.0)

AJCC = American Joint Committee on Cancer; BC = Breast cancer; LNM = Lymph node metastasis; NOS=Non-special

**Fig. 3:**
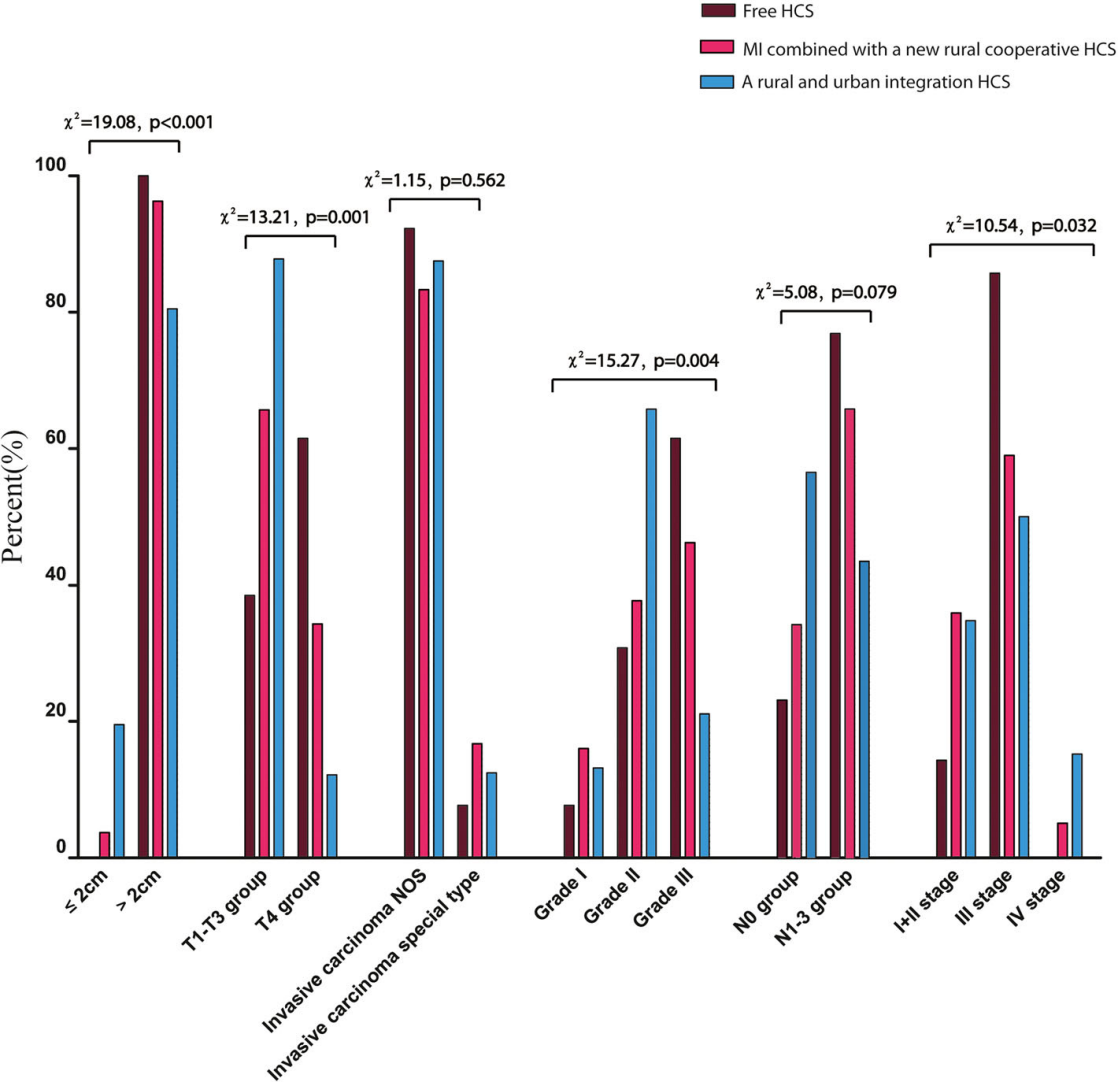
Comparison of characters of invasive breast cancer among different HCSs. Brown bars represent data in free HCS. Red bars represent data in MI combined with a new rural cooperative HCS. Blue bars represent data in a rural and urban integration HCS. The distribution of tumor size, T stage, pathologic grade as well as AJCC stage among different HCSs showed some differences, while the distribution of pathologic type and LNM showed no differences in the three different HCSs ^*^ significant difference < 0.05. Unknown cases are not shown in the figure.

*Relationship between clinicopathological characteristics of 270 Tibetan females with invasive BC from different HCSs.* Herein, we included only invasive BC since there were only 3 cases of DCIS. As shown in Fig. 3, Chi-square tests detected differences in the distribution of tumor size, T stage, pathological grade and AJCC stage among different HCSs (*P* < *0.05*).

Next, we used binary logistic regression for the correction of the results (Table 3). Consider the sample size, only T stage and AJCC (I + II &III) were analyzed. Binary logistic regression showed that the size of the T1–3 stage increased compared with T4 between a rural and urban integration HCS and free HCS (95%CI 0.046 to 0.852). the size of early stage (I + II) increased compared with stage III between these two HCSs (CI 0.019–0.633). The results were described in Table III. However, no differences between the pathological grades II and III among different HCSs, and grade I was used as reference (data not shown).

**Table 3:** The relationship between T stages and AJCC stage in Tibetan female with invasive carcinoma and healthcare system, census register and age group

Dependent factors	Covariates	sig	OR	95% C.I.
(T1–3 vs T4)	A rural and urban integration HCS vs Free HCS	0.030	0.198	(0.046, 0.852)
	MI combined with a new rural cooperative HCS vs Free HCS	0.123	0.382	(0.112,1.299)
	Census register (Urban residents vs farmers and herdsmen)	0.006	0.433	(0.236, 0.794)
	Age group (≤50 vs > 50)	0.565	1.183	(0.667, 2.100)
AJCC stage (I + II vs III)	A rural and urban integration HCS vs Free HCS	0.013	0.110	(0.019,0.633)
	MI combined with a new rural cooperative HCS vs Free HCS	0.075	0.233	(0.047,1.156)
	Census register (Urban residents vs farmers and herdsmen)	.000	.290	(0.160, 0.525)
	Age group (≤50 vs > 50)	.634	.867	(0.481, 1.562)

AJCC = American Joint Committee on Cancer; HCS = Health care system; MI = Medical insurance

## Discussion

In this study, for the first time, we detected the clinicopatholgical characteristics of Tibetan females with BC, whose level of HCS was lower in China as compared to the Han nationality. The results demonstrated that the Tibetan females with BC showed larger tumors (> 2 cm) with frequent T4 stage and later stage (more than a half of III stage). In the exploratory analysis, some clinicopathological characters of Tibetan women with BC may improve in different HCSs.

The current results showed that Tibetan females with BC presented more aggressive clinicopathologic characteristics in the three aspects as compared to the data of Han nationality [24–26]. The data of Han nationality were acquired from the seven districts in China or Shanghai, which represented the average or above level of HCS in Han nationality. Firstly, the proportion of small tumor size (≤2 cm) in Tibetans females with BC was lower than that of Hans (7.0% & 25.9%, respectively). Secondly, the proportion of N0 in Tibetan females with BC was lower than that in Hans (22.3% & 47.4%, respectively). Thirdly, the AJCC stage in Tibetan females with BC was usually later than that in Han female (50.5 and 47.3% in AJCC stages III and II, respectively). The differences in clinicopathologic characteristics between Tibetan and Han females with BC were similar to that of the other populations originated from HCS of different levels, such as African-American in the USA, India, and Kenya [14, 27–29]. These differences might be attributed to the following reasons. First, the difference in cancer awareness may be one of the reasons as shown previously [28, 30, 31]. TAR is an area about 1.22 million km^2^ consisting of 71 counties with an overall population density < 3/km^2^. The main population of this region comprised of farmers and herdsmen [16]. The number of medical technical personnel per 1000 people (unit, person) was 3.03, which was less than that in the East part of China (5.33) in 2012, that is one of the Han habitations [32]. A vast territory with a sparse population and low level of HCS may due to less cancer awareness, which might lead to the delay in early diagnosis. Second, the breast self-examination was lacking for a prolonged period and there was no breast screening until 2009 due to poor HCS. Since 2009, breast screening has been conducted according to the national guidelines of China. Briefly, Tibetan women underwent clinical breast examination, followed by B Ultrasound if abnormal, followed by mammography and biopsy [18]. Thus, clinical breast examination was the main screening tool in the low-income region as it was the most feasible model in these regions. The efficiency of clinical breast examination in the early detection was less than the mammography [13, 28, 29, 31, 33]. Third, other factors such as tumor biology, socioeconomic status, education, comorbidity, may also be the reasons for those differences mentioned before [9–11, 14, 15]. Thus, the prognosis of BC could be improved as follows. First, the awareness of BC should be improved through the internet, TV, lectures, visits, experiments, or topic discussions by focusing on the popularization of science on BC. Second, as skin invasion is common in Tibetan females with BC, they should be educated on the method of self-examination, focusing on the breast skin. Third, the doctors in the country should be trained for clinical breast examination. They are the first access for most patients with BC in TAR. The level of knowledge of these doctors might influence the level of diagnosis and treatment in BC.

Then we analyzed the influence of HCS on the characteristics of invasive BC in Tibet. We used HCS and census register as proxy to the SES in our study. The HCS in TAR has improved greatly (shown in Fig. 1) [16, 17, 21–23]. The maximal changes in the HCSs may lie in the health expenditures and subsidies by the government. TAR is an underdeveloped region in China, which needs government support, such as HCS. Figure 1 demonstrated great improvement in both aspects of subsidies and expenditure, although the raw data did not consider the influence of spending power, inflation, number of people served, age distribution of the population served, and competing priorities. However, the economic development in TAR was distinct, and the raw data might reflect the trend of subsidies by government and health expenditures in HCSs. Another characteristic of HCS in TAR was the relative stabilization of beds and medical technical personnel per 1000 patients, which differed from that in other underdeveloped regions such as Africa. Several physicians from the latter may migrate to developed countries due to various reasons when they become senior physicians [34]. In addition, several physicians of the Tibetan nationality were trained to serve the patients in their region. Thus, it can be confirmed that the HCS in TAR has been improved greatly in recent years. Also, the influence of HCS on the clinicopathologic characteristics of Tibetan females with BC using age group and census register as the two covariates was detected.

HCS may influence the clinicopathlogical characteristics. The improvement in HCSs can increase the detection rate of patients in an early stage and that in T1–3 tumor, which might be similar to that in Han nationality [25]. We also found the size of the larger tumor (> 2 cm) declined in free HCS, MI combined with a new rural cooperative HCS and a rural and urban integration HCS (data not shown). These may be three reasons. First, the screening of females with BC in TAR encompassed 18.3% (13/71) counties in farming and stockbreeding areas during 2009–2015, which not only helped discover patients of BC in the earlier stage, but also improved health education about cancer. The screening acquainted most Tibetan females in the farming and stockbreeding areas with BC for the first time. Consequently, a large number of BC patients sought hospital help instead of staying at home. Second, the improvement in HCS and traffic convenience allowed the patients to seek hospitalization. In the early time, patients from farming and stockbreeding areas in TAR may stay at home due to low income and traffic inconvenience. Nowadays, patients at a later stage and low income could also go to the hospital for help. Lasha, the political and economic center in TAR, represented the highest level of medical facilities in TAR and was the first preference for most Tibetan nationals. Third, the investment in health care made a marginal contribution [21]. However, the possibility of advanced (metastatic or recurrence BC/IV) stage showed some rising trend in a rural and urban integration HCS, the most recent, improved system. This was an interesting phenomenon. Although the proportion of the patients in early stage increased, we also saw 63% N positive and 27% T4 patients. One would expect a higher proportion of metastatic patients. Those were probably not diagnosed at first presentation due to lack of diagnostic facilties. For example, an inefficient clinical breast exam with respect to the detection of LNM as compared to mammography. Thus, the protocol of BC screening in TAR requires revision. In this revised protocol, health education, breast self-exam, and clinical breast exam were critical, followed by imaging test that mainly focused on the early detection of LNM, and pathological test as the highest diagnosis. Moreover, the duration of screening and the selection of high-risk populations necessitate further revision. As there were so many patients in III and IV stage, we thought the overall management in patients with advanced stage BC might be another key point in improving the prognosis of BC in the rural and urban integration HCS.

Nevertheless, the present study has some limitations. First, we selected Tibetan female patients with BC from TAR people’s hospital to represent the characteristics at presentation of the whole Tibetan population. The enrollment of BC patients in the selected hospital might be biased. However, the selection of this tertiary hospital was based on its ability in the standardization of diagnosis and treatment as well as the influence in TAR [16]. Another selection bias might be ascribed to the underestimation of the cases in the early years. TAR lies in the southwest frontier of China. The incidence of BC was 5.2/100000, based on the data of 2012 [25], indicating 52 cases annually, while the study reflects < 48.7% of the expected patients, especially for the early years of the hospital. Some patients did not come to the hospital. Second, some information was missing in some cases, which might influence the results. However, we selected relatively complete information for analysis. Third, the present study was a cross-sectional survey from 1973 to 2015 for 42 years. Several common factors such as the use of contraceptives or hormone replacement therapy, number of births, duration of breastfeeding, co-morbidities, and competing morbidities might also influence the results. However, TAR was an anoxic plateau, where only Tibetan nationals resided for centuries in a closed environment. The use of contraceptive and hormone replacement therapy was rare. Furthermore, each family had ≥2 children and all women choose breastfeeding. Altitude sickness was common in Tibetan nationality, especially in males, while this disease might affect slightly in Tibetan women. In addition, the government of TAR provides maximum support in cancer diagnosis and treatment nowadays, such as disease-specific reimbursement. Hence, most patients with BC seek a hospital for help. However, no definite evidence was available on co-morbidities and competing morbidities in the delayed diagnosis in Tibetan women with BC. However, an in-depth study is essential to confirm the findings of this study. In conclusion, the population features in this nationality showed relative stability of the results. Lastly, we did not consider the factors, such as the socio-economic status, altitude factors in different areas, and biomarkers, which might influence the present results. These differences will be analyzed in the following research. However, this was the first cohort study on Tibetan females with BC in TAR, and we adopted stringent selection and evaluation criteria. Taken together, the BC panorama in native Tibetan female, residents of high-altitude in a hypoxia environment, was described objectively.

## Conclusions

In this study, the characteristics of a Tibetan female with BC were analyzed, whose HCS level was relatively lower than those in China, and we also analyzed the influence of HCS on the characteristics at the time of presentation of the Tibetan female with BC. We have found that BC in Tibetan female showed aggressive clinicopathological characteristics at presentation, which might be at least partially attributed to poor HCS. In addition, some clinicopathological characteristics of Tibetan women with BC may improve in the rural and urban integration in HCS. The improved cancer awareness, early detection, and the overall management of patients with advanced stage BC might be valuable in the rural and urban integration HCS.

## Abbreviations

AJCC: American Joint Committee on Cancer
BC: breast cancer
CI: confidence interval
DCIS: ductal carcinoma in situ
HCS: healthcare system
LNM: lymph node metastasis
MI: medical insurance
NOS: non-special
TAR: Tibet Autonomous Region
